# The molecular mechanisms of chronic inflammation development

**DOI:** 10.3389/fimmu.2012.00323

**Published:** 2012-11-15

**Authors:** Masaaki Murakami, Toshio Hirano

**Affiliations:** ^1^Section of Developmental Immunology, JST-CREST, Graduate School of Frontier Biosciences, Graduate School of Medicine, iFReC, Osaka UniversityJapan; ^2^JST-CREST, Osaka UniversityJapan

Inflammation is critical for the development of many complex diseases and disorders including autoimmune diseases, metabolic syndrome, neurodegenerative diseases, cancers, and cardiovascular diseases. Inflammation comes in two types: chronic inflammation, which can be defined as a dysregulated form of inflammation, and acute inflammation, which can defined as a regulated form. Because of its special role in the aforementioned diseases, establishing methods to control chronic inflammation is important for developing cures and treatments against various diseases and disorders. One challenge to achieve this has been the ability to distinguish chronic and acute inflammation based on molecular biology diagnostics. Thus, this Research Topic is focused on articles that can shed new light on the molecular mechanisms responsible for the development of chronic inflammation and its related conditions.

This volume brings together 13 articles that are intended to provide a summary of some of the current thinking regarding the “molecular mechanism of chronic inflammation development.” The 13 articles are briefly described below.

The first (Chilton et al., 2012) and second articles (Shichita et al., 2012) present the roles of TLR-mediated signals in chronic inflammation. The authors focus a TLR4 ligand, lipid A, produced by Gram-negative bacteria and its variants to investigate how these compounds induce acute and chronic inflammation (Chilton et al., 2012) and post-ischemic inflammation in the brain induced by endogenous TLR ligands, high mobility group box 1 (HMGB1), and peroxiredoxin family proteins (Shichita et al., 2012). Both these exogenous and endogenous TLR ligands should be triggering factors for both acute as well as chronic inflammation.

The next three articles focus on T cells in the process of chronic inflammation. Huseby et al. (2012) review pathogenic CD8+ T cells in multiple sclerosis (MS), while many researchers including us paid much attention to pathogenic CD4+ T cells. Because it is known that lymphocytes in MS plaques are biased toward the CD8 lineage, the authors emphasize that understanding how CNS-reactive CD8 T cells escape tolerance induction and induce CNS autoimmunity is critical to our ability to propose and test new therapies for MS. It is possible, for example, that pathogenic CD8+ T cells might play a major role in chronic inflammation during the course of MS development. Mauro and Marelli-Berg (2012) report T cell immunity in cardiovascular metabolic disorders. They proposed that an altered metabolism in the cardiovascular system initially induced by macrophages and innate immunity can fuel chronic inflammation and subsequent migration of antigen-non-specific activated T cells to the affected site. Komatsu and Takayanagi (2012) address the synergistic activity of immune cells, particularly activated helper T cells and mesenchymal cells such as synovial fibroblasts in joints. They propose that mesenchymal cells, which interact with the activated T cells, are an important determinant in the development of chronic inflammation in the joints.

The next two articles show events in the later phase of chronic inflammation. Ueha et al. (2012) present cellular and molecular mechanisms of organ fibrosis and Rubin et al. (2012) address the relationship between IBD and colon cancer, which develops in the affected sites during chronic inflammation. The authors stress the formation of myofibroblasts mediated by activated helper T cells as well as cytokines such as TGFb for the development of chronic inflammation-mediated fibrosis (Ueha et al., 2012) and review the genetic basis of IBD, the genetic and cellular alterations associated with colitis-associated colon cancer, and the emerging role of the intestinal microbiota and other environmental factors.

The next three articles address the negative regulation of chronic inflammation by DHA metabolome, IL-10, and regulatory T cells (Tregs). Shinohara et al. (2012) present an overview of functional metabolomics that identified a new bioactive metabolome of docosahexaenoic acid (DHA) including their recent *in vivo* and *in vitro* data. They suggest that the metabolome of DHA might have protective and anti-inflammatory actions relevant even in humans. Kubo and Motomura (2012) review the transcriptional regulation of the anti-inflammatory cytokine, IL-10, particularly in adaptive immune cells such as activated helper T cells. Because IL-10 has a strong immune suppressive effect, it is possible that a reduced IL-10 transcription in the affected tissues can be a triggering factor for chronic inflammation. Fujio et al. (2012) discuss Tregs, which strongly inhibit chronic inflammation, by paying special attention to autoantibody production and autoantibody-mediated autoimmune diseases. They conclude that several kinds of Tregs have the potential to control autoimmune inflammation by suppressing both autoantibody production and the local chronic inflammatory responses that are induced by autoantibodies.

The next two articles focus on current ambiguities in immunology. Barnaba et al. (2012) review the various cell populations in the immune system. They hypothesize that they maintain a state of chronic low-level inflammation during persisting infections, and ultimately favor the species survival. Thus, dysregulation of low-level inflammation by enhancing harmful responses and/or reducing negative regulatory responses could develop into chronic inflammation. Prinz and Knobeloch (2012) present the good and bad of I-IFN with regards to immune responses and inflammation induction. I-IFN is associated with detrimental effects in Aicardi–Goutières syndrome (AGS), but at the same time is used as a standard therapeutic for the treatment of relapsing–remitting MS. The authors show mainly their own data for AGS, a severe disabling autoimmune inflammatory encephalopathy, and experimental autoimmune encephalomyelitis (EAE), a murine model of MS, to describe the roles of I-IFN during the development of chronic inflammation in the CNS.

The final report is ours (Murakami and Hirano, 2011). We have been studying the role of non-immune cells in the development of helper T cell-mediated autoimmune diseases. Although the NFκB-triggered positive-feedback-loop for IL-6-signaling (IL-6-amplifier) was originally discovered in mice to be a synergistic-activation signal that is activated following IL-17A and IL-6 stimulation in non-immune cells, results from disease models such as a rheumatoid arthritis, F759 mice, and EAE have shown that the IL-6-amplifier in fibroblasts, a type of endothelial cell, is activated by simultaneous stimulation of NFkB and STAT3 and locally induces chemokines and chronic inflammation. Indeed, we have proposed a four-step model for MHC class II-associated autoimmune diseases: (1) T cell activation regardless of antigen specificity; (2) local events inducing a tissue-specific accumulation of activated T cells; (3) transient activation of the IL-6 amplifier; and (4) enhanced sensitivity to cytokines in the target tissue. The interaction of these events results in chronic activation of the IL-6 amplifier and subsequent manifestation of autoimmune diseases. Thus, the IL-6 amplifier, which is chronically activated by these four events, is a critical regulator of chronic inflammations in tissue-specific MHC class II-associated autoimmune diseases.

As summarized above, the 13 articles present a range of data and issues that are under active investigation for understanding the molecular mechanism(s) of chronic inflammation, which is a general cause of various human diseases and disorders.
